# Strong contributions of local background climate to the cooling effect of urban green vegetation

**DOI:** 10.1038/s41598-018-25296-w

**Published:** 2018-05-01

**Authors:** Zhaowu Yu, Shaobin Xu, Yuhan Zhang, Gertrud Jørgensen, Henrik Vejre

**Affiliations:** Department of Geosciences and Natural Resource Management, Faculty of Science, University of Copenhagen, Copenhagen, 1958 Denmark

## Abstract

Utilization of urban green vegetation (UGV) has been recognized as a promising option to mitigate urban heat island (UHI) effect. While we still lack understanding of the contributions of local background climate to the cooling effect of UGV. Here we proposed and employed a cooling effect framework and selected eight typical cities located in Temperate Monsoon Climate (TMC) and Mediterranean Climate (MC) demonstrate that local climate condition largely affects the cooling effect of UGV. Specifically, we found increasing (artificial) rainfall and irrigation contribute to improving the cooling intensity of grassland in both climates, particularly in the hot-dry environment. The cities with high relative humidity would restrict the cooling effect of UGV. Increasing wind speed would significantly enhance the tree-covered while weakening the grass-covered UGVs’ cooling effect in MC cities. We also identified that, in order to achieve the most effective cooling with the smallest sized tree-covered UGV, the area of trees in both climate zones’ cities should generally be planned around 0.5 ha. The method and results enhance understanding of the cooling effect of UGVs on larger (climate) scales and provide important insights for UGV planning and management.

## Introduction

Urbanization significantly transformed the natural surfaces into impervious urban structures, which alter the materials, energy, radiation, and composition of the atmospheric structure in the near-surface layer^1–3^. During the urbanization, the modification of land surfaces, changes in surface material of buildings, and anthropogenic heat emission caused the urban heat island (UHI) effect, which results in the temperature in urban areas to be higher than the surrounding rural areas^4–6^. Additionally, climate change is increasingly recognized as an important factor that aggravates the UHI effect^7–9^. The UHI effect has led to many negative impacts, such as impairing air quality, increasing energy and water consumption, and damaging the urban residents’ health and well-being^4,10–12^.

Urban green vegetation (UGV) has increasingly been recognized as a promising option for the alleviation of adverse UHI effects by reducing thermal storage capacity and creating higher reflective surfaces (high albedo) that reduce the amount of absorbed solar radiance^3,13–16^. Meanwhile, the transpiration of UGVs and tree canopy shading can cool the environment by directly blocking solar radiance and preventing the heating of the land surface and air^13,17,18^. Considerable studies have found that the cooling effect of UGVs is dependent on the size of UGVs. Generally, though not always, UGV size is positively correlated with cooling intensity and the relationship tends to be non-linear, which confirms the law of diminishing marginal utility^10,19–21^. The cooling effect of UGVs is also associated with the growth condition and spatial heterogeneity of UGV landscape, and it contains the greenery (normally measured by normal difference vegetation index, NDVI), composition and configuration aspects that can significantly influence the cooling effect of UGVs^18,22–25^.

Additionally, studies have predicted and pointed out that the background climate conditions and the rain-heat relationship might influence the cooling effect of UGV, which need to be further considered^4,26,27^. For instance, Jansson *et al*.^28^ identified the cooling intensity of green space in continental climates ranged from 0.5 °C to 2.0 °C, while Rchid found that it can reach approximately 4.5 °C in hot desert climates^29^. Oliveira, *et al*.^30^ also supposed that the low cooling effect of green spaces was most likely associated with high humidity levels and reduced evapotranspiration. However, one of the status quos and challenges is that most of the related studies in this field are based on one specific city. The specific case-based studies do not provide a wider understanding of UGV cooling effects in the context of a global climate-changing world. Hence, Yu, *et al*.^10^ suggested that it is crucial to address a climate-zone-based study to further understanding of cooling effects from city level to global (climate) scale.

**Figure 1:**
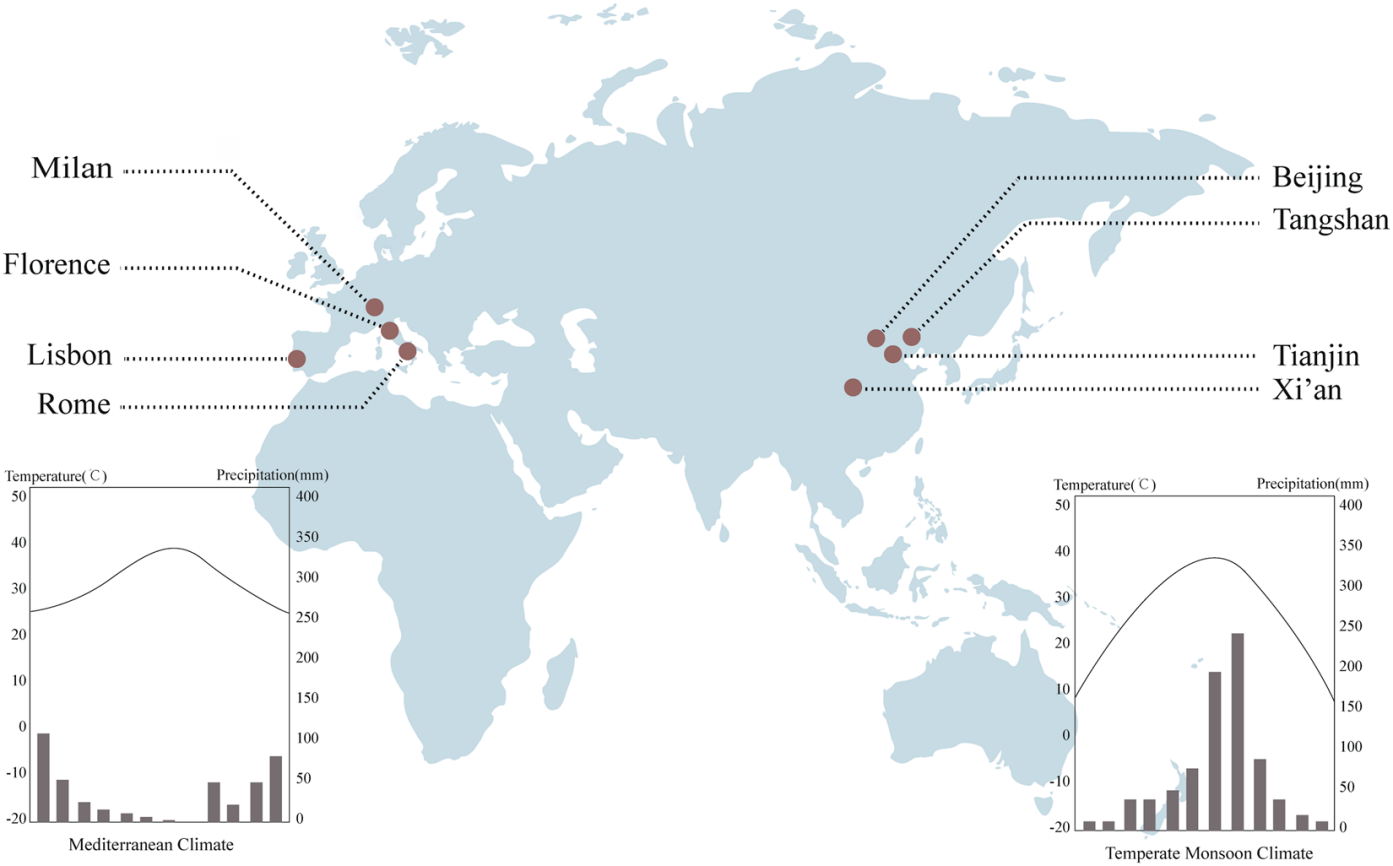
The location of selected study cities and the typical rain-heat relationship between TMC and MC zones. The TMC (Right) is characterized by the rain and heat over the same period, while the MC shows the opposite pattern (Left). Both climate zones have high summer temperatures and experience severe UHI effects. The TMC cities are Beijing, Tianjin, Tangshan, and Xi’an, and the MC cities are Rome, Florence, Milan, and Lisbon. We selected eight cities with similar climatic conditions within the two climate zones (Supplementary Information). The urbanized region within each city and relatively flat areas were chosen to avoid the influence of the terrain.

To address these insufficiencies and provide the climate zone scale implications for UGV management and planning. With the employment of the framework of the cooling effect (cooling intensity, extent, efficiency and threshold value of efficiency – TvoE) of UGV we proposed, we conducted a climate-zone-based study examining eight typical cities (Fig. 1) located in two climate zones: Temperate Monsoon Climate (TMC) and Mediterranean Climate (MC). The UGVs are classified into two categories: Tree-covered UGV (Tc-UGV) and Grass-covered UGV (Gc-UGV), then we explored the influence of different climate patterns on the cooling effect of UGV. We investigated and highlighting the strong contribution of local background climate (precipitation, relative humidity, and wind speed) to the cooling effect of UGV. Based on our findings, we suggest that global scale study on the cooling effect of UGV in a climate-changing world is critical. Besides, our finding can guide climate adaption based planning and decision-making in cities located in TMC and MC climate zone.

## Results and Discussion

### The cooling effect of UGV in TMC and MC zone cities

**Figure 2:**
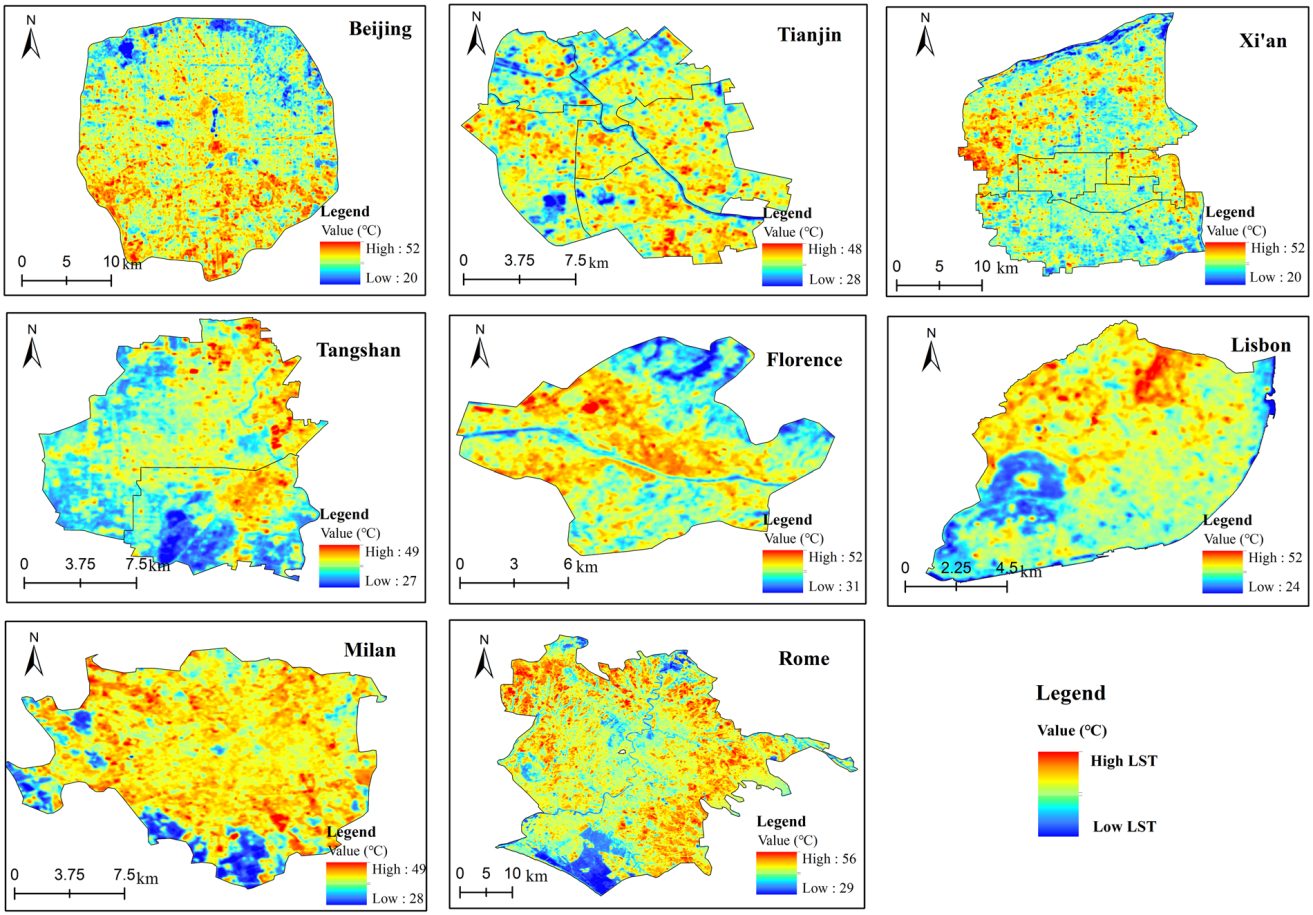
Land surface temperature retrieved by the Radiative Transfer Equation.

We calculated the LST by the Radiative Transfer Equation (Fig. 2), and we excluded the uncertain and anomalous data. We analyzed the effects of the area, shape, and NDVI of UGV and excluded these effects to ensure the accuracy of the results (Tables 1,2 and Supplementary Tables 1–6). We found the value of NDVI is significantly correlated with the cooling intensity and extent of Tree-covered UGV and Grass-covered UGV (Tables 1,2), which is in-line with previous studies^4,19,31^. Furthermore, we found the growth condition of UGV (the value of NDVI) in the MC cities is more important than in the TMC cities, especially for Tree-covered UGV. We suppose that the precipitation strongly contributes to this pattern. Additionally, the cooling intensity of Grass-covered UGV in the MC zone is more dependent on the vegetation growth than Tree-covered UGV, such as in the case of Milan (R^2^ = 0.608) and Rome (R^2^ = 0.605). This result in line with a desert city – Phoenix where the cooling effect of grassland was found to strongly rely on irrigation^32^. However, the influence of NDVI cannot be excluded (to next analysis) because the growth of vegetation is strongly dependent on the precipitation and humidity^27^, as well as strongly associated with climate conditions^18^.

**Table 1:** The relationship between three indexes (LSI, NDVI, area) and the cooling intensity of UGV.

City	Tree-covered UGV (Tc-UGV)			Grass-covered UGV (Gc-UGV)		
	LSI	NDVI	Area	LSI	NDVI	Area
Beijing	0.234^**^	0.378^**^	0.473^**^	0.157^**^	0.377^**^	0.381^**^
Tianjin	0.282^**^	0.258^**^	0.417^**^	0.114^*^	0.448^**^	0.436^**^
Tangshan	0.160^**^	0.248^**^	0.346^**^	0.282^**^	0.446^**^	0.540^**^
Xi’ an	0.204^**^	0.308^**^	0.413^**^	0.135^**^	0.396^**^	0.328^**^
Rome	0.207^**^	**0.508** ^**^	0.526^**^	−0.164^**^	**0.605** ^**^	0.173^**^
Florence	0.226^**^	**0.388** ^**^	0.527^**^	0.064	0.151	0.248^**^
Lisbon	0.095	**0.467** ^**^	0.562^**^	−0.082	0.328^**^	0.377^**^
Milan	0.145^**^	**0.312** ^**^	0.308^**^	0.017	**0.608** ^**^	0.468^*^

*Correlation is significant at the 0.05 level (2-tailed).

**Correlation is significant at the 0.01 level (2-tailed).

**Table 2:** The relationship between three indexes (LSI, NDVI, area) and the cooling extent of UGV.

City	Tree-covered UGV			Grass-covered UGV		
	LSI	NDVI	Area	LSI	NDVI	Area
Beijing	0.124^**^	0.196^**^	0.236^**^	0.750^**^	0.209^**^	0.191^**^
Tianjin	0.167^**^	0.165^**^	0.230^**^	0.065	0.307 ^**^	0.289^**^
Tangshan	0.640	0.144^**^	0.177^**^	0.127 ^*^	0.223^**^	0.257^**^
Xi’ an	0.116^**^	0.119^**^	0.191^**^	0.170	0.204^**^	0.065
Rome	0.042	0.170^**^	0.114^**^	−0.077	0.360^**^	−0.023
Florence	0.129^*^	0.198 ^**^	0.238^**^	−0.066	0.142	0.198^*^
Lisbon	0.028	0.266^**^	0.297^**^	0.061	0.196^*^	0.187
Milan	0.060	0.072	0.039	−0.181	0.263^**^	−0.048

*Correlation is significant at the 0.05 level (2-tailed).

**Correlation is significant at the 0.01 level (2-tailed).

Spearman’s Rho correlation analysis indicates the LSI (landscape shape index) of Tree-covered UGV is positively correlated with the cooling extent and intensity, which means more complex shape has a stronger cooling effect (Tables 1,2). This result is in line with previous studies^10,24,33^. For Grass-covered UGV, the correlation between cooling intensity and extent and LSI in TMC cities is significant and positive (Tables 1,2), while negatively correlated in MC cities. However, Yu *et al*.^10^ found that the positive/negative effects of shape depending on the size of UGV. When the size is less than 10 ha, a compact shape is better for cooling, and vice versa. General, the effects of LSI on cooling effect still has many controversies. We, therefore, hypothesize that the size of UGV, wind direction, and background climate can explain this pattern that needs to be further considered.

**Figure 3:**
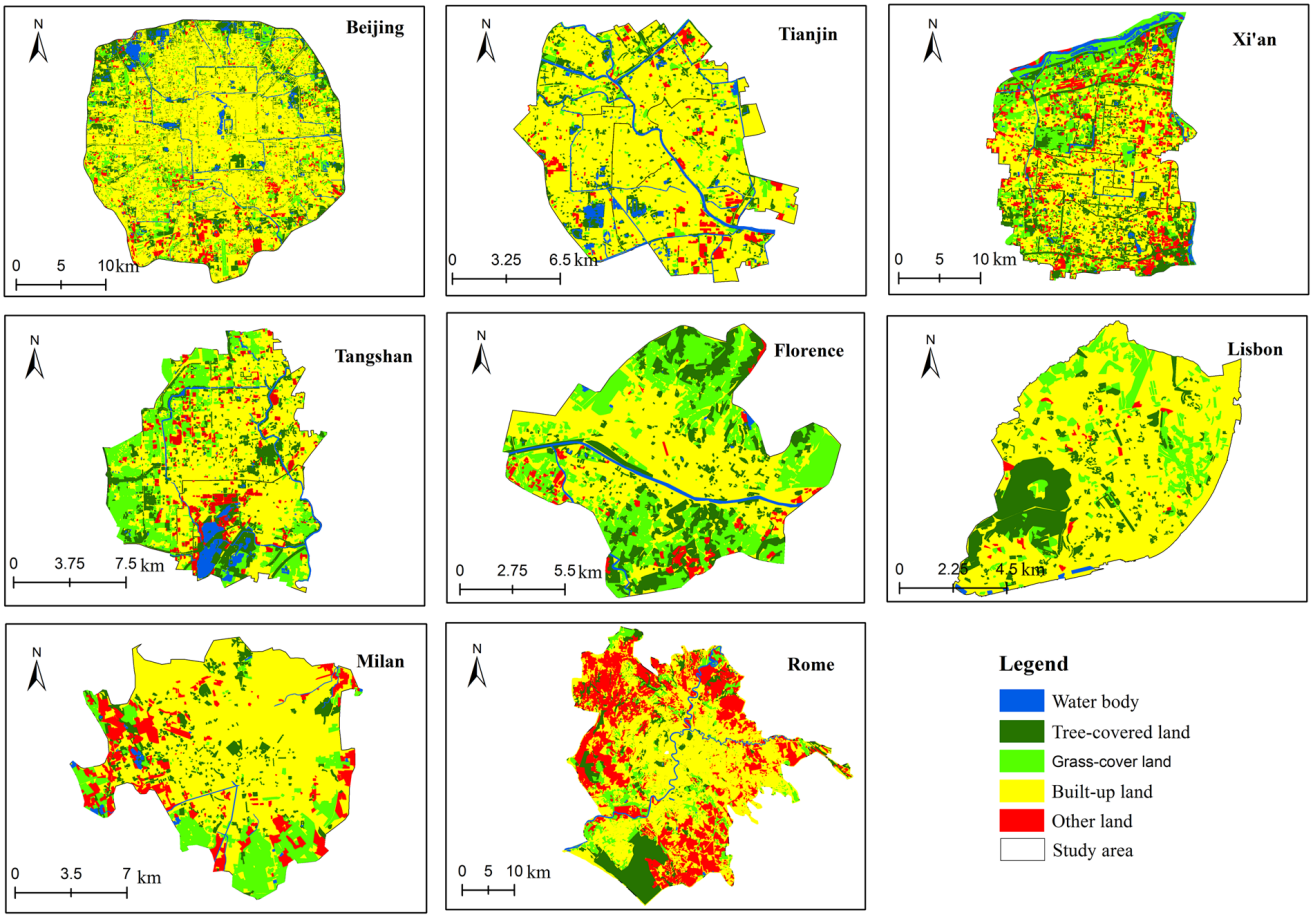
Land cover map in different cities.

We found strong correlations between the cooling intensity of UGV and size in both TMC and MC zones (Table 1), which is also in line with previous studies^4,13,23,34^. To the cooling extent of UGV, the TMC cities generally show a positive correlation, while the MC cities show a negative correlation (Table 2). Due to the index of the area is related to the landscape character of the UGV, which has no relationship with the climate conditions. Hence, it is critical to remove the effect of area on the cooling effect. We, afterward, excluded the effects of the area (Supplementary Tables 1–6) to proceed to obtain the adjusted result of the cooling effect of UGV. We found, generally, that the cooling intensity and extent of Tree-covered UGV does not have a big difference in either climate zone, while compared to the Grass-covered UGV, the Tree-covered UGV has a stronger cooling effect (Table 3). The adjusted cooling effect of UGV in Rome is significantly smaller, but the cooling intensity of Grass-covered UGV in Milan remains strong (Table 3 and Supplementary Table 7). This can be explained by the spatial pattern (Fig. 3) of UGV and precipitation (Rome and Milan are 0 mm and 57.91 mm, Supplementary Table 8). Regarding the TVoE (threshold value of efficiency), we found that larger Tree-covered UGV needs to be planned in MC cities as compared to TMC cities. We suppose that a larger Tree-covered UGV is better able to create a local microclimate (to increase evapotranspiration) in MC cities due to lack of summer precipitation.

**Table 3:** Adjusted results of the cooling intensity, extent, and TVoE for the Tree-covered UGV and Grass-covered UGV.

City	Tree-covered UGV			Grass-covered UGV		
	Cooling intensity (°C)	Cooling extent (m)	TVoE (ha)	Cooling extent (m)	Cooling Intensity (°C)	TVoE (ha)
Beijing	1.49	123	0.47	1.45	126	0.31
Tianjin	1.45	144	0.48	1.32	136	0.38
Tangshan	1.15	132	0.27	0.94	112	0.30
Xi’an	1.29	131	0.37	1.00	110	0.21
Rome	1.31	130	0.51	1.14	141	0.27
Florence	1.14	130	0.37	1.05	115	0.25
Lisbon	1.67	152	0.52	1.03	112	0.24
Milan	1.22	138	0.31	1.40	137	0.49

### Local background climate strongly affects the cooling effect of UGV

We found the strong contributions of local background climate to the cooling effect of UGV through this climate-zone-based study (Tables 4,5). This result is supported partly by previous literature review studies^4,24^. In terms of precipitation, the impact of precipitation on the cooling effect of Tree-covered UGV in both climate zones is not strong, while the Grass-covered UGV is highly dependent on the precipitation, especially for the MC cities. The strong correlation between the precipitation and cooling effect of Grass-covered UGV may be explained by the cooling mechanism of grassland – evapotranspiration, that the cooling effect of grassland in a dry area is highly dependent on the water supply^30,32^. In addition, the water supply is also associated with the growth condition of grassland, which is directly related to their NDVI values. The relationship between the cooling effect of Grass-covered UGV and their NDVI value is similar to this pattern (Tables 1,2). The reason why the cooling effect of Tree-covered UGV is not highly correlated with the precipitation is: (1) evapotranspiration is not the only cooling mechanism for the tree (e.g., others include shade effect and convection efficiency)^15,23,35,36^; (2) a certain area of trees can produce a microclimate that can maintain the humidity. Furthermore, the TVoE and precipitation in the MC zone are also highly correlated (R^2^ = −0.65), which means that the areas with higher rainfall need a greater size of Tree-covered UGV. This pattern is seen in Milan (TVoE and precipitation are 0.49 ha and 57.91 mm) and Florence (TVoE and precipitation are 0.25 ha and 11.94 mm).

**Table 4:** The relationships between the cooling effect of Tree-covered UGV and the temperature, precipitation, relative humidity.

Climate Zone	Cooling effect component	Temperature	Precipitation	Relative humidity	Wind speed
Temperate Monsoon	Cooling intensity	0.259	0.341	−**0.752**	0.126
	Cooling extent	−0.174	−0.024	0.002	0.335
	TVoE	0.245	0.230	−**0.641**	0.256
Mediterranean	Cooling intensity	−0.013	−0.090	−0.047	**0.875**
	Cooling extent	−0.296	0.014	0.021	**0.568**
	TVoE	0.312	−**0.646**	−0.401	**0.643**

The p-value test was not applied in this section due to the limited sample size (n = 8).

**Table 5:** The relationships between the cooling effect of Grass-covered UGV and the temperature, precipitation, relative humidity.

Climate Zone	Cooling effect component	Temperature	Precipitation	Relative humidity	Wind speed
Temperate Monsoon	Cooling intensity	0.104	**0.627**	−**0.942**	0.004
	Cooling extent	0.003	0.475	−**0.739**	0.080
	TVoE	−0.291	0.555	−**0.507**	−0.004
Mediterranean	Cooling intensity	−0.038	**0.791**	−0.004	−0.493
	Cooling extent	**0.328**	**0.818**	−0.480	−0.358
	TVoE	−0.044	**0.721**	−0.032	−0.209

The p-value test was not applied in this section due to the limited sample size (n = 8).

We found the relative humidity is negatively associated with the cooling effect of UGV in the TMC zone (Tables 4,5). Similar results are found in the MC zone, yet the strength of the correlation is much weaker than that of the TMC zone. Negative correlations between relative humidity and the cooling effect of UGV indicate that increased relative humidity will result in a lower cooling effect^1,30^. This pattern could be related to the cooling mechanism of evapotranspiration. Jung^37^ and Wang^32^ explained that increasing relative humidity would result in a decrease the evapotranspiration rate, as well as the cooling effect of UGV. Compared with the MC and TMC zones, the humidity of the TMC zone is higher than that of the MC zone, which results in the evapotranspiration of UGV in TMC zone being sensitive to changes in relative humidity. This is also why the correlation in TMC zone is stronger than that of MC zone.

We found that the wind speed is strongly positively correlated with the cooling effect of Tree-covered UGV in the MC zone, while negatively correlated with the Grass-covered UGV (Tables 4,5). In the TMC zone, the wind speed is also positively associated with the cooling effect of Tree-covered UGV, while the correlation is weaker than that of the MC zone. This result may contribute to the evapotranspiration and heat convection of UGVs. Dimoudi and Nikolopoulou^38^ and Gunawardena *et al*.^13^ revealed that the evapotranspiration rate and convective heat transfer coefficient were higher with higher wind speed conditions when the air temperature exceeded 25 °C. The increase in wind speed would result in a decrease in leaf temperature and help to transfer the cooling effect to the surrounding area. However, when the temperature exceeds a certain point, the effect of evapotranspiration decreases. In MC cities, the significant temperature difference between the Tree-covered UGV and surrounding environment can be easily recognized (Supplementary Tables 9,10). For Tree-covered UGV in MC cities, the wind speed would contribute to transfer cooler air (convective heat transfer) to their surrounding environment. The reason for the negative correlation of Grass-covered UGV would be the evapotranspiration decreasing under the condition of high temperature. The relatively weak correlation in TMC cities might be explained by the presence of intensely compact urban structures with many high-rise buildings. Therefore, the aerodynamics and microscale wind condition in these cities are more complex than in MC cities.

### Limitations and further study

Although the strong contributions of local background climate to the cooling effect of UGV was found, some limitations still need to be mentioned. Firstly, the effects of microclimate environment around the specific UGV patch. In this study, due to the purpose of research (we mainly emphasis the general pattern and relationship on a larger scale in this study, so we properly ignored some potential factors) and the accessibility of data, we do not take the potentially influential factors, i.e., wind direction^13,39^, convection efficiency^13^, building height^10^ and land cover/use pattern^20^ around the specific UGV patch, into consideration. These factors are considered to affect the cooling effect of UGV, which needs further consideration in the next. Secondly, we did not consider the aerodynamic characteristics of UGV. Studies have demonstrated that aerodynamic roughness of UGV can influence the wind speed and direction^1,4,40^. For instance, Kent *et al*.^40^ found where vegetation is taller and occupies a greater amount of space, wind speeds may be slowed by up to a factor of three. Thirdly, multiple temporal-spatial scales should be considered. As a result of the limit of Landsat image acquisition, it is difficult to find multiple high quality (9 level) images (summer is the raining season in TMC zone) to do the study, hence we just selected one timestamp images. Even the results based on one timestamp image is acceptable in this field^16,21,34^ and can represent the situation in this region (time) to some extent^18,33,41^, more comparable cases cities and multiple time images are also necessary to develop more reliable results in the further study.

### Conclusion remarks

Previous studies paid much attention to investigating the effect of composition and configuration of UGVs on the cooling effect in specific case city, while we still do not fully understand the contributions of local background climate to the cooling effect of UGV^10,20^. Using the framework of cooling effect and selected eight cities located in the TMC and MC zones. Theoretically, we revealed the strong contributes of local background climate to the cooling effect of UGV, which enhances understanding of the cooling effect of UGVs on larger scales (from composition and configuration to climate zone), as well as in the context of climate change. Besides, our finding points out when the air becomes increasingly saturated, the cooling effect of UGV would weaken. However, in the MC zone, the opposite effect of relative humidity would occur when their values reach a certain point. We also found that increasing wind speed would significantly improve the cooling effect of Tree-covered UGV in the MC zone while impairing the cooling effect of Grass-covered UGV.

Practically, our findings provide important insights and principles for UGV planning and management in terms of the cities located in the TMC and MC zones. (1) Grass-covered UGV in the MC zone should increase (artificial) precipitation and irrigation to enhance the cooling effect in MC cities. For example, building a fountain on the existing grassland would help mitigate the UHI effect significantly in MC cities. (2) Tree-covered UGV is the best solution for mitigating and adapting UHI effect that can have a stronger cooling effect in both TMC and MC zones. (3) In order to achieve the strongest cooling effect with the smallest size of Tree-covered UGV, it should generally be planned around 0.5 ha. (4) Wind speed can significantly reduce temperature around the Tree-covered UGV. Hence, the air passage needs more consideration in urban planning, especially in MC cities and a large, highly urbanized city (e.g., Beijing) with many high-rise buildings and complex urban structures.

## Method

### Data collection and processing

The datasets of this study include three categories: (1) Landsat 8 (TIRS) remote sensing (RS) data; (2) Historical Google Earth images; (3) meteorological data. For remote sensing images, each image was acquired during summer daytime hours and was cloud-free over the study area, therefore land surface temperature (LST) would not be affected (Supplementary Table 11). The historical high-resolution Google Earth images (summer 2015 were used for land cover classification. The climate data for each city came from the corresponding meteorological station. The station numbers of each city are: 54511 (Beijing), 54527 (Tianjin), 54534 (Tangshan), 57131 (Xi’an), 162350 (Rome), 161700 (Florence), 85790 (Lisbon), 160800 (Milan). The historical climate data of the European cities are available online (www.en.tutiempo.net), and the climate data of the Chinese cities were acquired from the China Meteorological Bureau. These climate data include three parameters: precipitation, relative humidity, and wind speed. Relative humidity and wind speed refer to daily weather conditions. Climate data parameters were retrieved for time periods corresponding to acquisition dates of the Landsat 8 images.

### Land surface temperature retrieving

The land surface temperature in each city (Fig. 2) was calculated using the Radiative Transfer Equation (RTE). This method used band 10 thermal radiance, which was obtained from the Landsat 8 thermal infrared sensor (TIRS). The RTE method involves estimating atmospheric effects on surface thermal radiation, then subtracting it from the total amount of heat radiation observed by satellite sensors. Then, the intensity of thermal radiation can be converted into the LST^42,43^.

In practice, the RTE method can express the apparent radiance (L_λ_) received by a sensor. The atmospheric downward radiance (L_atm_,_i_↓), the upward radiance (L_atm,i_↑), and the transmissivity (τ) can hence be estimated (http://atmcorr.gsfc.nasa.gov/). Finally, the ground radiance, B(T_s_) in Eq. (1), can be calculated using the given land surface emissivity (ε). The LST can be determined using Eq. (2):

1 $${{\rm{L}}}_{{\rm{\lambda }}}=[{\rm{\varepsilon }}B({{\rm{T}}}_{{\rm{S}}})+(1-{\rm{\varepsilon }})\,{{\rm{L}}}_{{\rm{atm}},{\rm{i}}}\downarrow ]{\rm{\tau }}+{{\rm{L}}}_{{\rm{atm}},{\rm{i}}}\,\uparrow \,,$$

where ε is the surface emissivity, T_S_ is the LST, B(Ts) is the ground radiance, and τ is atmospheric transmittance. Therefore, according to Plank’s law, B(Ts) can be expressed as Eq. (2). Then, the LST can be calculated using Eq. (3):

2 $${\rm{B}}({{\rm{T}}}_{{\rm{S}}})=[{{\rm{L}}}_{{\rm{\lambda }}}-{{\rm{L}}}_{{\rm{atm}},{\rm{i}}}\,\uparrow -{\rm{\tau }}(1-{\rm{\varepsilon }})\,{{\rm{L}}}_{{\rm{atm}},{\rm{i}}}\downarrow ]/{\rm{\tau }}{\rm{\varepsilon }}$$

3 $${{\rm{T}}}_{{\rm{s}}}={{\rm{K}}}_{2}/\mathrm{ln}\,({{\rm{K}}}_{1}/{\rm{B}}({{\rm{T}}}_{{\rm{S}}})+1)$$

For Landsat-8 TIRS band 10, K_1_ = 774.89 (mW m^−2^s·r^−1^μm^−1^), K_2_ = 1321.08 K.

### Land cover mapping

Previous studies have provided several methods for land cover mapping, such as object-based, supervised, and unsupervised image classification^12,16^. However, all of these methods can be influenced by many uncertainties that can affect the accuracy of the result^44^. In order to obtain a more accurate result, we used the method of visual interpretation to map the land cover classification of the case cities. With the help of Google Earth Pro software and its historical image database, we manually delineated five types of land cover in 2015: built-up land, tree-covered land (urban forest), grass-covered land (grassland), water body, and other land. The other land category refers to bare land covered with sand and bare soil. The water body refers to the lakes, rivers, and ditches in the city. The tree-covered land and the grass-covered land refer to urban green vegetation. Specifically, when the percentage of tree canopy exceeds 30% in a green patch, it would be classified into the category of the tree-covered land. When canopy coverage is less than 30%, it would be regarded as grassland. Finally, we created a land cover map of each city (Fig. 3).

### Definition of the cooling effect of urban green vegetation

**Figure 4:**
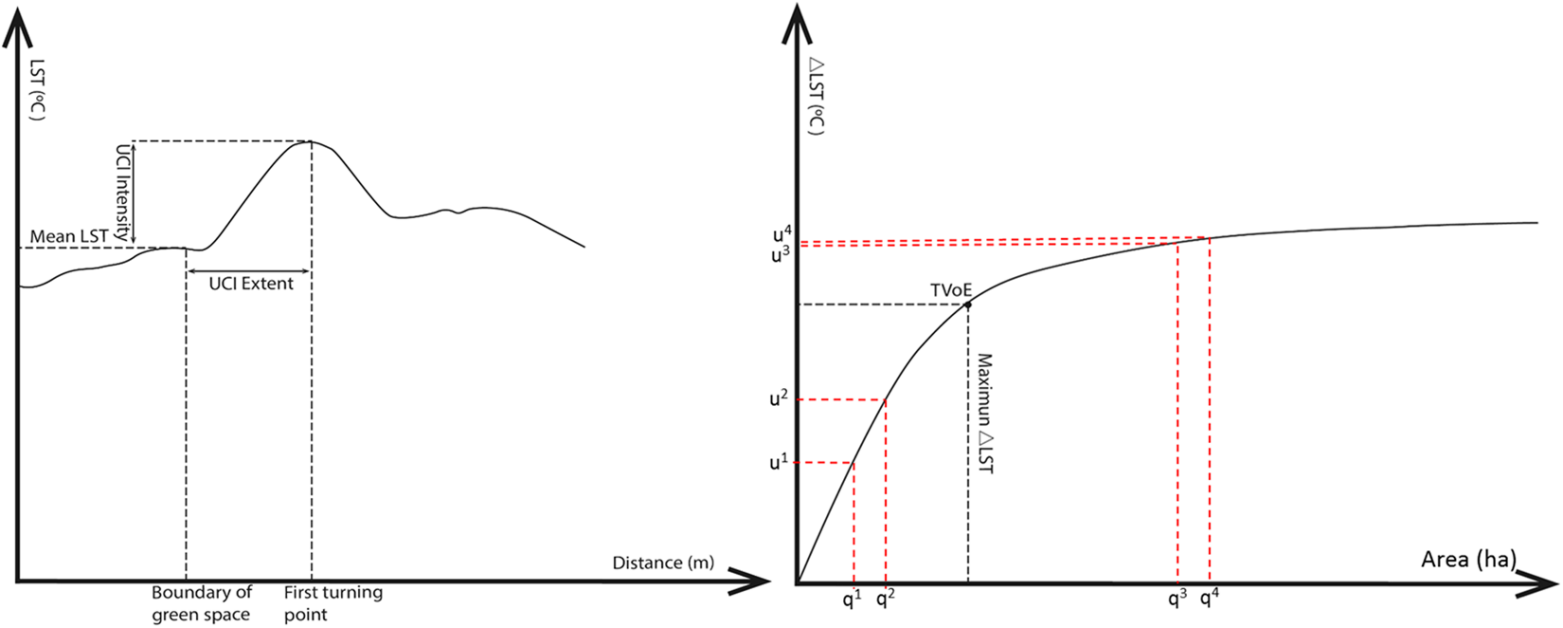
The conceptual curve of urban cooling island (UCI) extent, intensity, efficiency, and TVoE. The q^1^ to q^2^ equal q^3^ to q^4^, u^2^–u^1^ greater than u^4^–u^3^.

Previous studies have proposed some definitions to calculate and express the cooling effect of urban green vegetation^31,45^. For example, Hamada and Ohta^41^ defined the cooling intensity as the temperature difference between green patches and the built-up environment (LST_b-g_). Lin *et al*.^21^ suggested that the calculation of the cooling extent of a park is just like the calculation lake basin. In this study, we employed the four-part framework proposed in our previous study on the cooling effect of UGV: cooling extent, cooling intensity, cooling efficiency, and the threshold value of efficiency (TVoE)^10^. Specifically, the cooling effect of UGV is expressed as the temperature difference between the UGVs and the surrounding urban area. The maximum cooling extent of a UGV is expressed as the distance between the edge of the UGV and the first turning point of temperature drop compared with the UGV temperature. This turning point is the maximum ΔLST and defined as the cooling intensity of a UGV (Fig. 4). The cooling efficiency is expressed as a logarithmic curve between the area of each UGV and its maximum ΔLST. The cooling efficiency curve conforms a logarithmic function, which means calculating the reciprocal of the logarithmic function can get the TVoE point of UGV (x:y equals 1:1 in this reciprocal function, greater than this constant, x > y, which means the cooling efficiency of UGV is decreasing). Accordingly, we can calculate the TVoE value to achieve the maximum cooling effect while using the smallest UGV area.

### Spatial and statistical analyses

We excluded UGVs connected with water bodies and with areas < 900 m^2^ to ensure accuracy. Finally, we identified Tree-covered UGV (4947, 757, 1603, 912, 312, 93, 762, 277), and Grass-covered UGV patches (1799, 349, 658, 417, 113, 330, 265, 120) in Beijing, Tianjin, Xi’an, Tangshan, Lisbon, Milan, Rome, and Florence, respectively. Based on the results of previous studies that the cooling extent of a UGV is generally less than 500 m^19,21,24^, we used multiple buffer zones (16 buffers in each patch, 30–480 m) around the outline of each green patch in ArcGIS 10.2. We then calculate the cooling effect of each patch. Account for the effects of area, shape, and NDVI, we analyzed the effects and excluded them. The landscape shape index was used to identify the shape effect^46^:

$$LSI=Perimeter/2\sqrt{\pi \times Area},$$

where LSI equals 1 for a circle and 1.13 for a square. The NDVI value ranged from −1 to 1, and it described the growth and the coverage amount of vegetation. It is calculated as:

$$NDVI=Band3-Band2/Band3+Band2,$$

where band data were obtained from the Landsat 8 images^31,45^. After excluding the influencing factors, linear regression was used to investigate the relationship between the background climate conditions and the cooling effect of UGV.

## Electronic supplementary material

Supplementary Information
