# Supplementary material for: Strong contributions of local background climate to the cooling effect of urban green vegetation
Source: Sci Rep. 2018 May 1;8:6798. doi: 10.1038/s41598-018-25296-w (PMC5931616; doi:10.1038/s41598-018-25296-w)
Supplement: Supplementary file 1 — Supplementary Information [file 41598_2018_25296_MOESM1_ESM.docx]

**Strong contributions of local background climate to the cooling effect of urban green vegetation**

**Zhaowu Yu^[[1]](#footnote-1)^*, Shaobin Xu^1^, Yuhan Zhang^1^,** **Gertrud Jørgensen^1^, Henrik Vejre^1^**

# **Supplementary Information**

## **1. Case selection**

We selected the temperate monsoon climate (TMC) and the Mediterranean climate (MC) for our study since they have different climate characters and occur in the same latitude band. The TMC is characterized by the rain and heat over the same period, while the MC show the different pattern. (Figure 1). Both climate zones have high summer temperatures and experience severe UHI effects (Hoag 2015; Sun et al. 2014; Zhou et al. 2017). In TMCs, 60 – 70% of the annual rainfall (500-1000 mm) occurs during summer due to the influence of monsoon wind, which is a typical climate phenomenon in Asia. In MCs, 60-70% of the annual rainfall (300-1000 mm) occurs during winter; summers are hot and very dry. The relationship between rain and heat in these climates are common to many regions throughout the world and thus provides a good opportunity to explore the contributions of the background climate on the cooling effect of UGV.

We selected eight cities with similar climatic conditions within the two climate zones (Fig. 1): TMC [Beijing (39.90°N, 116.41°E), Tianjin (39.34°N, 117.36°E), Tangshan (39.63°N, 118.18°E), and Xi’an (34.34°N, 108.94°E)] and MC [Rome (41.90°N, 12.50°E), Florence (43.77°N, 11.26°E), Milan (45.46°N, 9.19°E), and Lisbon (38.72°N, 9.14°W)]. These cities are characterized by compact urban structures and high population densities. We studied the urbanized region within each city and chose relatively flat areas to avoid the influence of the terrain. For instance, the municipality of Beijing (16412 km²) has 16 districts, and consists of urban, suburban and rural districts. We selected the Fifth-Ring Road (highly urbanized area) as our case area. For more detailed information on our case cities.

## **2. Exclude the effects of NDVI, Shape and Area**

Based on the results of Spearman’s Rho correlation analysis (Tab.1), we can find that the effect of the NDVI and area is generally strong. Therefore, we need to exclude the potential influence to further the analysis on contributions of background climate to the cooling effect of UGV. However, the influence of NDVI cannot be excluded because the growth of vegetation is strongly dependent on the precipitation and humidity (Spronken-Smith and Oke 1998) and strongly associated with climate conditions. The index of area is related to the spatial character of the UGVs, which has no relationship with the climate conditions. Hence, it is critical to remove the effect of area on the cooling effect.

**Supplementary Table. 1.** Mean area of Tc-UGV after classification

| Area (ha) | Beijing | Tianjin | Tangshan | Xi' an |  | Rome | Florence | Lisbon | Milan |
| --- | --- | --- | --- | --- | --- | --- | --- | --- | --- |
| 0-0.2 | 0.149 | 0.151 | 0.146 | 0.142 |  | 0.144 | 0.163 | 0.136 | 0.173 |
| 0.2-0.4 | 0.296 | 0.289 | 0.288 | 0.297 |  | 0.329 | 0.309 | 0.282 | 0.308 |
| 0.4-0.6 | 0.490 | 0.499 | 0.496 | 0.496 |  | 0.507 | 0.483 | 0.488 | 0.506 |
| 0.6-0.8 | 0.693 | 0.698 | 0.697 | 0.680 |  | 0.701 | 0.685 | 0.713 | 0.698 |
| 0.8-1.0 | 0.891 | 0.887 | 0.884 | 0.902 |  | 0.906 | 0.892 | 0.912 | 0.892 |
| 1.0-2.0 | 1.412 | 1.406 | 1.402 | 1.399 |  | 1.493 | 1.441 | 1.486 | 1.444 |
| 2.0-4.0 | 2.834 | 2.706 | 2.901 | 2.821 |  | 2.899 | 2.894 | 2.941 | 2.835 |
| 4.0-8.0 | 5.647 | 5.179 | 5.409 | 5.616 |  | 5.735 | 5.518 | 5.627 | 5.470 |
| 8.0-10.0 | 8.854 | 8.836 | 9.178 | 8.759 |  | 8.907 | 8.930 | 8.766 | 8.349 |
| >10.0 | 29.291 | 19.599 | 27.614 | 31.009 |  | 79.348 | 30.901 | 116.090 | 25.430 |

**Supplementary Table. 2.** Mean area of Gc-UGV after classification

| Area (ha) | Beijing | Tianjin | Tangshan | Xi' an |  | Rome | Florence | Lisbon | Milan |
| --- | --- | --- | --- | --- | --- | --- | --- | --- | --- |
| 0-0.2 | 0.148 | 0.150 | 0.147 | 0.149 |  | 0.000 | 0.026 | 0.114 | 0.000 |
| 0.2-0.4 | 0.292 | 0.291 | 0.282 | 0.303 |  | 0.259 | 0.322 | 0.303 | 0.311 |
| 0.4-0.6 | 0.480 | 0.493 | 0.493 | 0.492 |  | 0.496 | 0.509 | 0.555 | 0.499 |
| 0.6-0.8 | 0.679 | 0.680 | 0.703 | 0.704 |  | 0.682 | 0.691 | 0.720 | 0.737 |
| 0.8-1.0 | 0.896 | 0.899 | 0.888 | 0.899 |  | 0.898 | 0.889 | 0.888 | 0.928 |
| 1.0-2.0 | 1.432 | 1.437 | 1.478 | 1.428 |  | 1.454 | 1.442 | 1.546 | 1.610 |
| 2.0-4.0 | 2.844 | 2.836 | 2.870 | 2.815 |  | 2.952 | 2.894 | 2.930 | 2.814 |
| 4.0-8.0 | 5.494 | 5.335 | 5.609 | 5.552 |  | 5.755 | 5.720 | 5.443 | 5.928 |
| 8.0-10.0 | 8.924 | 8.627 | 8.856 | 8.854 |  | 8.841 | 9.053 | 9.325 | 8.819 |
| >10.0 | 27.332 | 19.041 | 46.189 | 30.464 |  | 46.233 | 65.199 | 16.504 | 51.829 |

**Supplementary Table. 3.** Mean cooling extent of Tc-UGV after classification

| Area (ha) | Beijing | Tianjin | Tangshan | Xi' an |  | Rome | Florence | Lisbon | Milan |
| --- | --- | --- | --- | --- | --- | --- | --- | --- | --- |
| 0-0.2 | 91.190 | 105.714 | 96.885 | 98.257 |  | 156.000 | 138.000 | 90.000 | 115.000 |
| 0.2-0.4 | 104.545 | 108.932 | 115.588 | 115.714 |  | 128.000 | 107.143 | 116.000 | 134.082 |
| 0.4-0.6 | 115.722 | 125.164 | 109.429 | 126.398 |  | 142.000 | 77.143 | 126.429 | 153.158 |
| 0.6-0.8 | 121.556 | 118.554 | 124.286 | 120.694 |  | 116.000 | 156.000 | 161.739 | 130.588 |
| 0.8-1.0 | 116.729 | 130.962 | 142.500 | 146.870 |  | 121.111 | 140.000 | 165.652 | 123.529 |
| 1.0-2.0 | 144.031 | 146.139 | 137.500 | 131.863 |  | 134.494 | 134.667 | 176.170 | 136.324 |
| 2.0-4.0 | 137.155 | 172.642 | 159.231 | 146.420 |  | 128.976 | 102.188 | 155.556 | 148.269 |
| 4.0-8.0 | 142.466 | 156.818 | 150.000 | 159.281 |  | 140.276 | 153.659 | 132.353 | 139.355 |
| 8.0-10.0 | 141.000 | 240.000 | 158.571 | 141.429 |  | 106.429 | 162.500 | 247.500 | 165.000 |

**Supplementary Table. 4.** Mean cooling extent of Gc-UGV after classification

| Area (ha) | Beijing | Tianjin | Tangshan | Xi' an |  | Rome | Florence | Lisbon | Milan |
| --- | --- | --- | --- | --- | --- | --- | --- | --- | --- |
| 0.2-0.4 | 105.864 | 120.000 | 95.373 | 100.526 |  | 126.000 | 45.000 | 105.000 | 180.000 |
| 0.4-0.6 | 106.604 | 112.826 | 92.791 | 105.667 |  | 180.000 | 127.500 | 120.000 | 126.000 |
| 0.6-0.8 | 132.273 | 130.800 | 110.690 | 116.757 |  | 135.000 | 126.000 | 72.000 | 150.000 |
| 0.8-1.0 | 130.633 | 125.357 | 124.000 | 117.447 |  | 150.000 | 145.714 | 130.000 | 90.000 |
| 1.0-2.0 | 128.654 | 142.000 | 105.818 | 109.930 |  | 130.000 | 103.043 | 107.647 | 132.857 |
| 2.0-4.0 | 137.868 | 156.207 | 118.824 | 111.667 |  | 141.951 | 96.667 | 110.526 | 150.000 |
| 4.0-8.0 | 139.468 | 167.647 | 130.286 | 113.196 |  | 112.500 | 131.053 | 150.000 | 140.000 |
| 8.0-10.0 | 132.000 | 140.000 | 120.000 | 108.333 |  | 158.824 | 150.000 | 105.000 | 131.250 |

**Supplementary Table. 5.** Mean cooling intensity of Tc-UGV after classification.

| Area (ha) | Beijing | Tianjin | Tangshan | Xi' an | Rome | Florence | Lisbon | Milan |
| --- | --- | --- | --- | --- | --- | --- | --- | --- |
| 0-0.2 | 0.683 | 0.684 | 0.566 | 0.694 | 0.797 | 0.527 | 0.614 | 0.789 |
| 0.2-0.4 | 0.891 | 0.737 | 0.736 | 0.841 | 1.034 | 0.749 | 0.807 | 0.882 |
| 0.4-0.6 | 1.086 | 1.011 | 0.822 | 1.026 | 1.130 | 0.600 | 0.900 | 1.134 |
| 0.6-0.8 | 1.231 | 1.035 | 0.790 | 1.040 | 0.954 | 1.157 | 1.348 | 0.933 |
| 0.8-1.0 | 1.243 | 1.164 | 0.990 | 1.244 | 1.044 | 1.056 | 1.700 | 1.022 |
| 1.0-2.0 | 1.678 | 1.495 | 1.151 | 1.176 | 1.415 | 1.371 | 1.730 | 1.137 |
| 2.0-4.0 | 1.875 | 1.968 | 1.588 | 1.659 | 1.528 | 1.295 | 2.063 | 1.265 |
| 4.0-8.0 | 2.262 | 2.034 | 1.622 | 1.989 | 1.968 | 1.732 | 2.272 | 1.530 |
| 8.0-10.0 | 2.465 | 2.915 | 2.100 | 1.944 | 1.920 | 1.753 | 3.548 | 2.280 |

**Supplementary Table. 6.** Mean cooling intensity of Gc-UGV after classification.

| Area (ha) | Beijing | Tianjin | Tangshan | Xi' an | Rome | Florence | Lisbon | Milan |
| --- | --- | --- | --- | --- | --- | --- | --- | --- |
| 0.2-0.4 | 0.837 | 0.834 | 0.571 | 0.637 | 0.787 | 0.383 | 0.650 | 0.748 |
| 0.4-0.6 | 0.941 | 0.982 | 0.522 | 0.742 | 0.939 | 0.865 | 0.736 | 0.523 |
| 0.6-0.8 | 1.257 | 1.034 | 0.763 | 0.865 | 0.766 | 1.007 | 0.594 | 1.489 |
| 0.8-1.0 | 1.403 | 1.027 | 0.829 | 0.909 | 1.115 | 1.080 | 1.179 | 0.990 |
| 1.0-2.0 | 1.323 | 1.321 | 0.863 | 0.933 | 1.065 | 0.718 | 1.099 | 1.241 |
| 2.0-4.0 | 1.656 | 1.712 | 1.132 | 1.061 | 1.231 | 0.803 | 1.164 | 1.400 |
| 4.0-8.0 | 1.847 | 1.922 | 1.414 | 1.295 | 1.483 | 1.089 | 1.578 | 2.728 |
| 8.0-10.0 | 2.357 | 1.723 | 1.420 | 1.552 | 1.721 | 2.478 | 1.276 | 2.095 |

## **3. Results of the cooling intensity, extent, and TVoE for tree-covered and grass-covered UGV**

**Supplementary Table. 7.** Results of the cooling intensity, extent, and TVoE for tree-covered and grass-covered UGV

|  | Tc-UGV | | | Gc-UGV | | |
| --- | --- | --- | --- | --- | --- | --- |
| City | **Cooling intensity (**°C**)** | **Cooling extent (m)** | **TVoE (ha)** | **Cooling**  **Intensity (**°**C)** | **Cooling extent (m)** | **TVoE (ha)** |
| Beijing | 1.34 | 121.05 | 0.45 | 1.21 | 119.79 | 0.32 |
| Tianjin | 1.11 | 126.77 | 0.41 | 0.99 | 108.82 | 0.37 |
| Tangshan | 0.99 | 125.26 | 0.37 | 1.08 | 125.01 | 0.30 |
| Xi’an | 1.26 | 130.82 | 0.39 | 0.98 | 109.09 | 0.22 |
|  |  |  |  |  |  |  |
| Rome | 2.03 | 137.25 | 0.50 | 1.66 | 125.38 | 0.38 |
| Florence | 1.32 | 130.43 | 0.40 | 0.81 | 103.78 | 0.22 |
| Lisbon | 1.33 | 135.34 | 0.52 | 1.10 | 111.19 | 0.27 |
| Milan | 1.20 | 138.91 | 0.52 | 2.15 | 129.42 | 0.30 |

## **4. Climate data**

The climate conditions are expressed by four parameters: temperature (obtained from average LST of study areas), precipitation, relative humidity, and wind speed. Climate data for eight cities was derived from corresponding meteorological stations. The p-value test was not applied in this section due to the limited sample size (n=8).

**Supplementary Table. 8.** Climate data from eight cities (derived from corresponding meteorological station).

|  | Relative humidity (%) | Wind speed (m/s) | Precipitation (mm) | Mean LST  (°C) |
| --- | --- | --- | --- | --- |
| Beijing | 50 | 2.3 | 82.60 | 39.931 |
| Tianjin | 54 | 2.9 | 77.40 | 37.238 |
| Tangshan | 65 | 2.1 | 74.50 | 34.932 |
| Xi' an | 68 | 2.9 | 65.20 | 40.867 |
| Rome | 38 | 2.4 | 0.00 | 43.024 |
| Florence | 57 | 2.1 | 11.94 | 39.823 |
| Lisbon | 50 | 3.8 | 10.67 | 39.353 |
| Milan | 51 | 1.6 | 57.91 | 39.096 |

## **5. Temperature difference**

The △LST_b-g_ means the temperature difference between UGV patches and the built-up environment (LST_b-g_) (Hamada and Ohta 2010; Wong et al. 2016).

**Supplementary Table. 9.** The basic information of Tc-UGV

| City | Research number | Total Area (ha) | Mean Area (ha) | Mean LST (˚C) | △LST_b-g_  (˚C) |
| --- | --- | --- | --- | --- | --- |
| Beijing | 3787 | 10705.779 | 2.472 | 37.341 | 3.376 |
| Tianjin | 758 | 1046.844 | 1.149 | 34.932 | 2.753 |
| Tangshan | 656 | 1892.157 | 2.538 | 32.956 | 3.305 |
| Xi`an | 1603 | 5203.672 | 3.016 | 38.688 | 2.724 |
|  |  |  |  |  |  |
| Rome | 772 | 22532.836 | 28.145 | 38.668 | 4.643 |
| Florence | 279 | 1675.060 | 5.636 | 36.935 | 4.213 |
| Lisbon | 317 | 1344.229 | 4.068 | 35.338 | 4.626 |
| Milan | 330 | 995.476 | 2.813 | 37.360 | 2.532 |

**Supplementary Table. 10.** The basic information of Gc-UGV

| City | Research number | Total Area (ha) | Mean Area (ha) | Mean LST (˚C) | △LST_B-G_  (˚C) |
| --- | --- | --- | --- | --- | --- |
| Beijing | 1281 | 3623.362 | 2.418 | 38.459 | 2.258 |
| Tianjin | 408 | 3363.854 | 7.772 | 35.480 | 2.205 |
| Tangshan | 353 | 625.705 | 1.516 | 32.759 | 3.502 |
| Xi`an | 916 | 8462.437 | 5.395 | 40.266 | 1.146 |
|  |  |  |  |  | 0.000 |
| Rome | 262 | 13264.249 | 22.839 | 42.362 | 0.949 |
| Florence | 135 | 3088.267 | 7.916 | 39.326 | 1.822 |
| Lisbon | 109 | 861.355 | 4.349 | 40.864 | -0.900 |
| Milan | 86 | 2468.884 | 21.140 | 35.595 | 4.297 |

## **6. Landsat data, LST and land cover mapping**

These Landsat data obtained from the official website of the U.S. Geological Survey (https://www.usgs.gov/). The remote sensing images of each city were selected on the condition that (1) the acquisition date of the image comes from the summer daytime (June, July, and August) in 2015; (2) Less cloud cover above the study area in the corresponding acquisition date. The detail information of Landsat 8 images for each city were presented in Table S1. It is important to stress that the cloud cover in this table refers to the whole satellite image, which include a large area. This value cannot reveal the atmospheric condition above these cities. Actually, there is no cloud cover in any study area.

**Supplementary Table. 11.**  Basic information of Landsat 8 satellite imagery

| City | Landsat Scene Identifier | Satellite Acquisition Date | GMT Time | Local Time | Cloud Cover (%) |
| --- | --- | --- | --- | --- | --- |
| Beijing | LC81230322015234LGN01 | August 22, 2015 | 02:53 | 10:53 | 33.33 |
| Tianjin | LC81230332015234LGN01 | August 22, 2015 | 02:53 | 10:53 | 5.85 |
| Tangshan | LC81220322015227LGN01 | August 15, 2015 | 02:46 | 10:46 | 0.07 |
| Xi'an | LC81270362015214LGN01 | August 2, 2015 | 03:19 | 11:19 | 21.63 |
|  |  |  |  |  |  |
| Rome | LC81910312015198LGN01 | July 17, 2015 | 09:52 | 10:53 | 2.02 |
| Florence | LC81920302015205LGN01 | July 24, 2015 | 09:58 | 10:58 | 1.6 |
| Lisbon | LC82040332015177LGN01 | June 26, 2015 | 11:13 | 12:13 | 0.08 |
| Milan | LC81940282015219LGN01 | August 7, 2015 | 10:10 | 11:10 | 8.32 |

Previous studies have provided several methods for land cover mapping, such as object-based, supervised, and unsupervised image classification (Peng et al. 2016; Sun and Chen 2017). However, all of these methods can be influenced by many uncertainties that can affect the accuracy of the result (Estoque et al. 2017). In order to obtain a more accurate result, we use the method of visual interpretation to map the land cover classification of the case cities. With the help of Google Earth Pro software and its historical image database, we manually delineated five types of land cover in 2015: built-up land, tree-covered land (urban forest), grass-covered land (grassland), water body, and other land. The other land category refers to bare land covered with sand and bare soil. The water body refers to the lakes, rivers, and ditches in the city. The Tc-UGV and the Gc-UGV refer to green vegetation, which is the main focus of this study. Specifically, when the percentage of tree canopy exceeds 30% in a green patch, it would be classified into the category of the Tc-UGV. When canopy coverage is less than 30%, it would be regarded as Gc-UGV. Finally, we created a land cover map of each city (Figure. 2).

## **Supplementary References:**

Estoque, R.C., Murayama, Y., & Myint, S.W. (2017). Effects of landscape composition and pattern on land surface temperature: An urban heat island study in the megacities of Southeast Asia. *Science of The Total Environment, 577*, 349-359

Hamada, S., & Ohta, T. (2010). Seasonal variations in the cooling effect of urban green areas on surrounding urban areas. *Urban Forestry & Urban Greening, 9*, 15-24

Hoag, H. (2015). How cities can beat the heat. *Nature, 524*, 402

Peng, J., Xie, P., Liu, Y., & Ma, J. (2016). Urban thermal environment dynamics and associated landscape pattern factors: A case study in the Beijing metropolitan region. *Remote Sensing of Environment, 173*, 145-155

Spronken-Smith, R., & Oke, T. (1998). The thermal regime of urban parks in two cities with different summer climates. *International journal of remote sensing, 19*, 2085-2104

Sun, R., & Chen, L. (2017). Effects of green space dynamics on urban heat islands: Mitigation and diversification. *Ecosystem Services, 23*, 38-46

Sun, Y., Zhang, X., Zwiers, F.W., Song, L., Wan, H., Hu, T., Yin, H., & Ren, G. (2014). Rapid increase in the risk of extreme summer heat in Eastern China. *Nature Clim. Change, 4*, 1082-1085

Wong, P.P.-Y., Lai, P.-C., Low, C.-T., Chen, S., & Hart, M. (2016). The impact of environmental and human factors on urban heat and microclimate variability. *Building and environment, 95*, 199-208

Zhou, W., Wang, J., & Cadenasso, M.L. (2017). Effects of the spatial configuration of trees on urban heat mitigation: A comparative study. *Remote Sensing of Environment, 195*, 1-12

1. 1 Department of Geosciences and Natural Resource Management, Faculty of Science, University of Copenhagen, Copenhagen, 1958, Denmark. Correspondence and requests for materials should be addressed to Z.Y. (email: zhyu@ign.ku.dk) [↑](#footnote-ref-1)
